# Supplementary figures and images for: NADPH-dependent thioredoxin reductase C plays a role in nonhost disease resistance against Pseudomonas syringae pathogens by regulating chloroplast-generated reactive oxygen species
Source: PeerJ. 2016 Apr 26;4:e1938. doi: 10.7717/peerj.1938 (PMC4860297; doi:10.7717/peerj.1938)

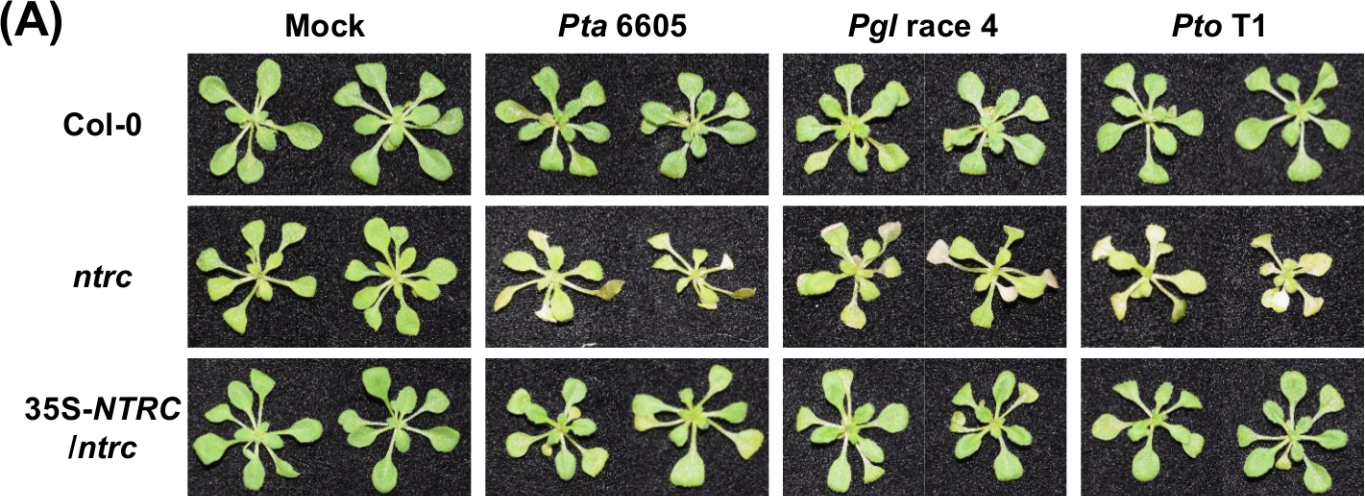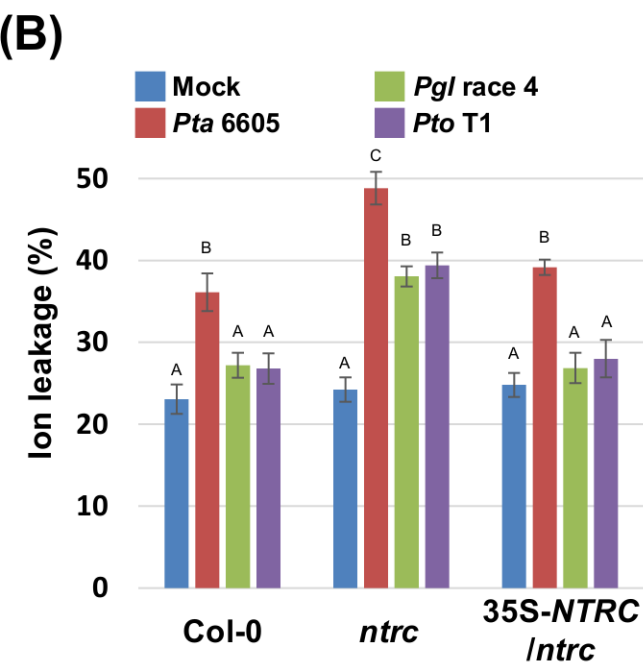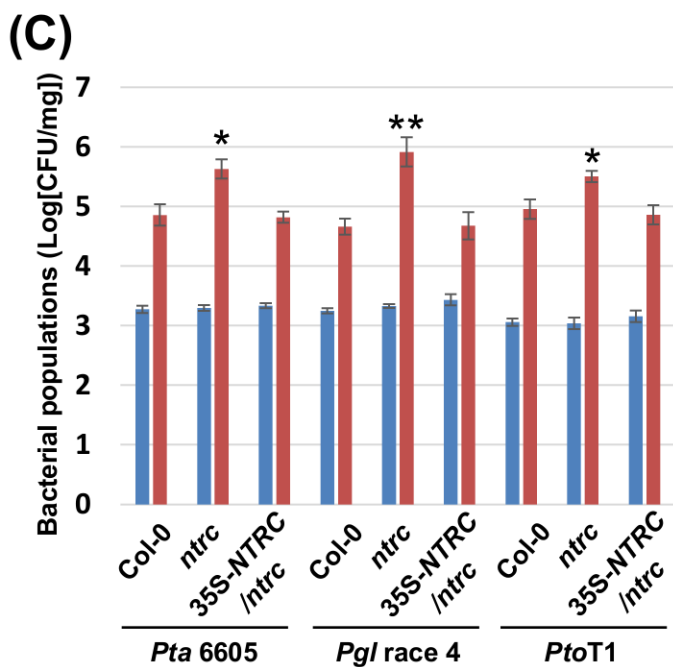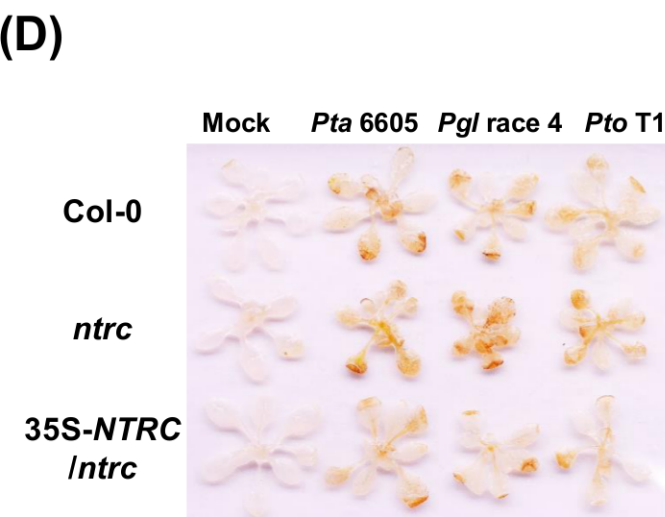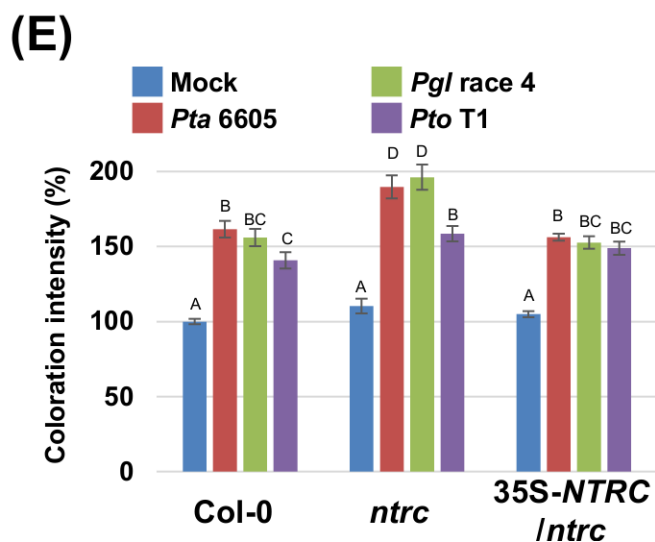

Supplement: Figure S1 — (A) Response of the Arabidopsis thaliana wild-type Col-0, ntrc mutant, and the complemented line (35S-NTRC/ntrc) to nonhost Pseudomonas syringae pathogens. Arabidopsis seedlings were individually flood-inoculated with nonhost pathogens including Pseudomonas syringae pv. tabaci (P. s. pv. tabaci), P. syringae pv. glycinea (P. s. pv. glycinea), and P. syringae pv. tomato T1 (P. s. pv. tomato T1) at a concentration of 5 × 10^6^ CFU/ml. Photographs were taken at 2 dpi. (B) Ion leakage from the Arabidopsis thaliana wild-type Col-0, ntrc mutant, and the complemented line (35S-NTRC/ntrc) flooded with water (mock), or Pseudomonas syringae pv. tabaci (P. s. pv. tabaci), P. syringae pv. glycinea (P. s. pv. glycinea), or P. syringae pv. tomato T1 (P. s. pv. tomato T1) at a concentration of 5 × 10^6^ CFU/ml. Samples were collected at 2 dpi. Bars show the percentage of total ions. Vertical bars indicate the standard error for three biological replicates. Statistically significant differences are noted as different alphabet characters based on ANOVA (p < 0.05). (C) Bacterial populations of Pseudomonas syringae pv. tabaci (P. s. pv. tabaci), P. syringae pv. glycinea (P. s. pv. glycinea), and P. syringae pv. tomato T1 (P. s. pv. tomato T1) in the Arabidopsis wild-type Col-0, ntrc mutant, and the complement line (35S-NTRC/ntrc). Bacterial populations were quantified at 0 and 2 dpi. Vertical bars indicate the standard errors for three independent experiments. Asterisks indicate a significant difference from the wild-type Col-0 in a t-test (∗ = p < 0.05, ∗∗ = p < 0.01). (D) Hydrogen peroxide production in the Arabidopsis wild-type Col-0, ntrc mutant, and the complemented line (35S-NTRC/ntrc) in response to P. syringae pv. tabaci, P. syringae pv. glycinea, and P. syringae pv. tomato T1 at a concentration of 5 × 10^6^ CFU/ml. ROS were visualized by staining hydrogen peroxide using 3,3′-diaminobenzidine at 2 dpi. (E) The intensity of DAB staining shown in panel D is quantified and expressed as [file peerj-04-1938-s001.pdf]

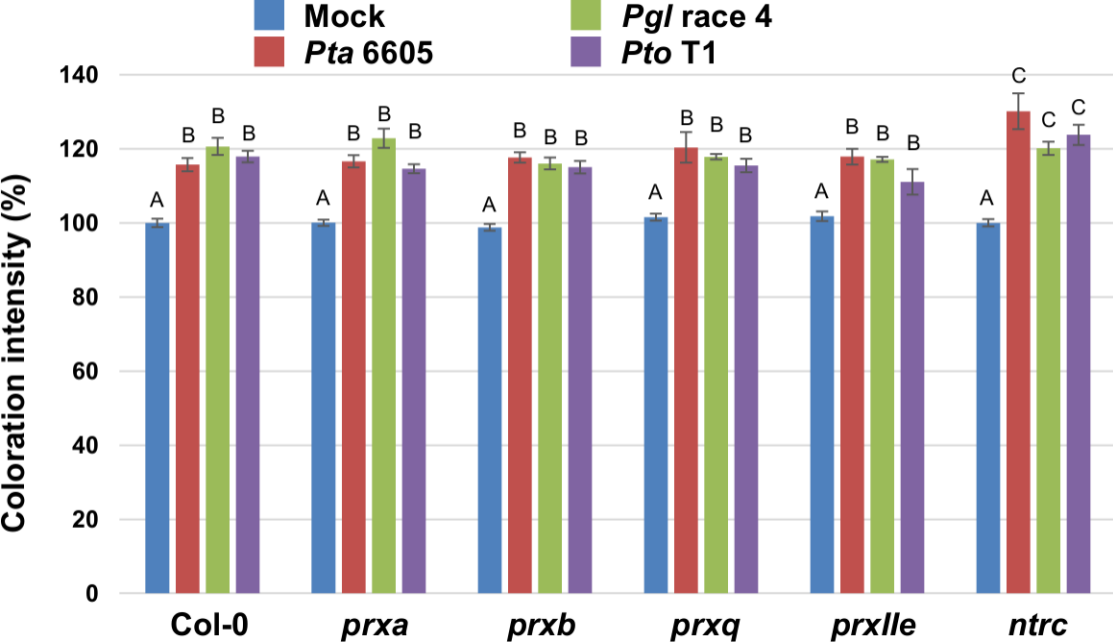

Supplement: Figure S2 — ROS were visualized by staining hydrogen peroxide using 3,3′-diaminobenzidine at 1 dpi. The intensity of DAB staining is quantified and expressed as a percentage of coloration, where the color intensity of mock-treated wild-type Col-0 leaves was set at 100%. Vertical bars indicate the standard error for 10 leaves. Statistically significant differences are noted as different alphabet characters based on ANOVA (p < 0.05). [file peerj-04-1938-s002.pdf]

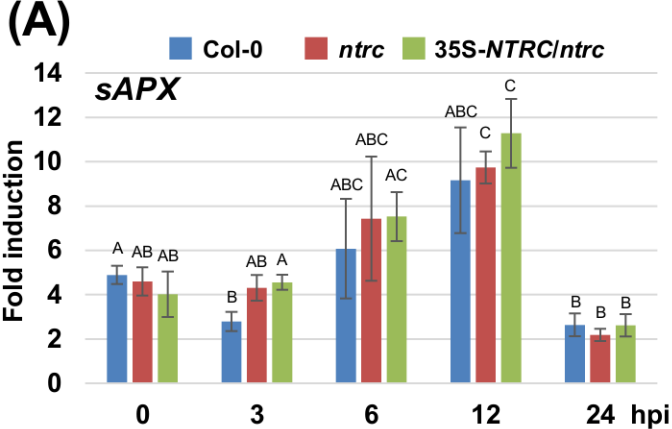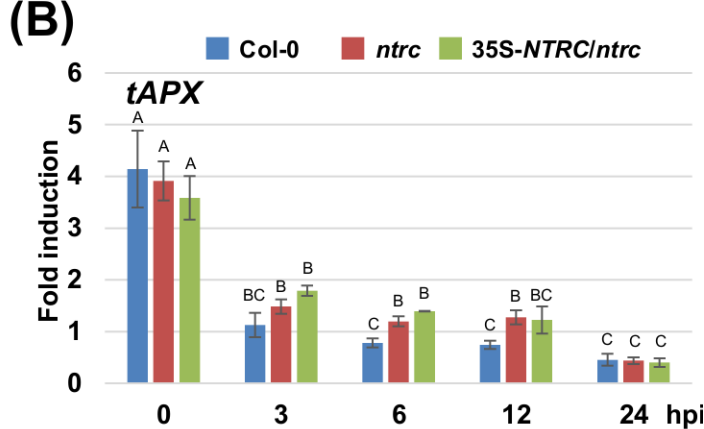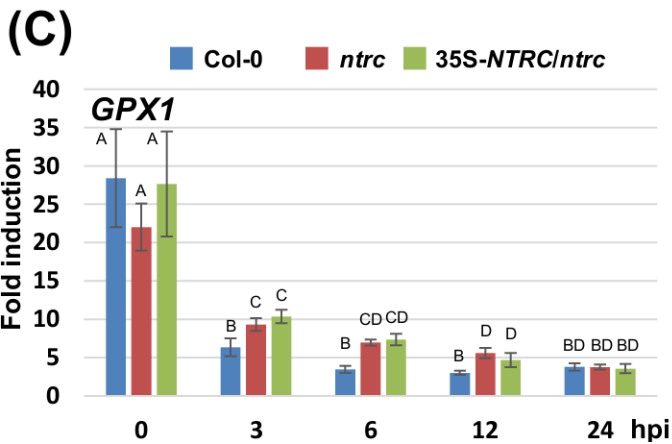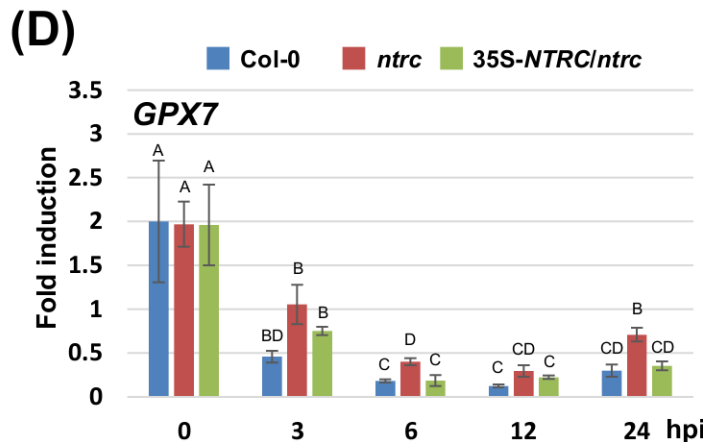

Supplement: Figure S3 — The expression profiles of the genes related to ROS detoxification including STROMAL ASCORBATE PEROXIDASE (A), THYLAKOIDAL ASCORBATE PEROXIDASE (B), GLUTATHIONE PEROXIDASE 1 (C), and GLUTATHIONE PEROXIDASE 7 (D) were obtained at 3, 6, 12, and 24 h after inoculation with the nonhost pathogen Pseudomonas syringae pv. tabaci. Two-week-old Arabidopsis Col-0, ntrc, and 35S-NTRC/ntrc plants were treated with water as a mock control (Mock), or inoculated with nonhost P. syringae pv. tabaci at a concentration of 5 × 10^6^ CFU/ml. The expression of genes was evaluated by RT-qPCR with gene-specific primer sets (Table S1). The values represent the relative induction compared to the expression of UBQ1. The figures are from a representative experiment and were repeated at least 3 times with similar results. Vertical bars indicate the standard error for three biological replicates. Statistically significant differences are noted as different alphabet characters based on ANOVA (p < 0.05). [file peerj-04-1938-s003.pdf]
