# Supplementary material for: NADPH-dependent thioredoxin reductase C plays a role in nonhost disease resistance against Pseudomonas syringae pathogens by regulating chloroplast-generated reactive oxygen species
Source: PeerJ. 2016 Apr 26;4:e1938. doi: 10.7717/peerj.1938 (PMC4860297; doi:10.7717/peerj.1938)
Supplement: Table S1 [file peerj-04-1938-s004.docx]

**Supplementary Table S1. The list of primers used in this study.**

| Gene | Locus Number | FW primer | RV primer |
| --- | --- | --- | --- |
| *UBQ1* | AT3G52590 | GGCCTTGTATAATCCCTGATGAATAAG | AAAGAGATAACAGGAACGGAAACATAGT |
| *ICS1* | AT1G74710 | CATTGATCTATGCGGGGACA | TGGACAAAAGCTCGTACCTGA |
| *PR1* | AT2G14610 | GCCGTGAACATGTGGGTTAG | GGCACATCCGAGTCTCACTG |
| *LOX2* | AT3G45140 | AGCTTATTAGCGCCAAGACTGACCA | GCCACCCATGACTCACATGTAAACG |
| *OPR3* | AT2G06050 | AGCAAGTTGTGGAAGCAGTTCACG | TAAAGCCCGAGGTTTCGGGTACTT |
| *MYC2* | AT1G32640 | TCATACGACGGTTGCCAGAA | AGCAACGTTTACAAGCTTTGATTG |
| *GRX480* | AT1G28480 | ACCACAGAGCCCCAACTTC | GGCGGTCCTTGAGATTGAT |
| *ANAC019* | AT1G52890 | GCATCTCGTCGCTCAG | CTCGACTTCCTCCTCCG |
| *JAZ3* | AT3G17860 | TCCTCCAGGTTCCATTGTAGGGACT | CGGCGAAAAGACTTGAGGCATAGA |
| *sAPX* | AT4G08390 | GGACACCAGAGTGGCTGAAGTTTG | GTGCCCCCATTAGATAACGATACCC |
| *tAPX* | AT1G77490 | TCCCGAGGGCATAGTCATTGAAAAC | GAGAGTGGACAAGAGGACCAAAACG |
| *GPX1* | AT2G25080 | TCTCGTCCCTTCCTTGAAATTCTCC | GCAACATCCTTCCCATCAATGTCC |
| *GPX7* | AT4G31870 | TTTTCTCGGCCCATCATTGAGATTC | CGCAACGTTAACGATCAACAAAGG |
